# The competency of the novel through‐the‐scope suture device for gastric mucosal defects: In vivo study in a porcine model (with video)

**DOI:** 10.1002/deo2.70037

**Published:** 2024-11-12

**Authors:** Mamoru Ito, Akira Dobashi, Takanori Tominaga, Toshiki Futakuchi, Naoto Tamai, Machi Suka, Kazuki Sumiyama

**Affiliations:** ^1^ Department of Endoscopy The Jikei University School of Medicine Tokyo Japan; ^2^ Department of Gastroenterology and Hepatology The Jikei University School of Medicine Tokyo Japan; ^3^ Department of Public Health and Environmental Medicine The Jikei University School of Medicine Tokyo Japan

**Keywords:** endoscopic closure, endoscopic resection, gastric mucosal defects, learning curve, suture device

## Abstract

**Objectives:**

Endoscopic closures of mucosal defects following endoscopic resection can be challenging and time‐consuming. The novel through‐the‐scope suture device has demonstrated acceptable closure times, but its learning curve is still unknown. This study aims to evaluate the number of cases required to achieve competency in this device.

**Methods:**

Two endoscopists participated; a novice with less than 400 experiences in upper gastrointestinal endoscopy and an expert with over 500 experiences in endoscopic submucosal dissection. Neither endoscopist had previous exposure to the device. In four porcine models, 24 gastric mucosal defects, each 2–4 cm in diameter, were created by endoscopic mucosal resection with ligation. Each endoscopist performed endoscopic closure for 12 mucosal defects with a single through‐the‐scope suture device per lesion. The primary endpoint was the number of cases needed to reach competency, defined as achieving a procedure time below the average closure time reported in the literature. Secondary endpoints included procedure time, complete closure success rates, and incidence of adverse events.

**Results:**

The mean defect size was 2.9 (±0.2) cm. Competency was achieved after six cases in the expert and seven cases in the novice. The median closure time was 9.0 (interquartile range [IQR]: 6.0–11.0) min for the expert and 8.0 (IQR: 6.2–9.7) min for the novice (*p* = 0.862). Complete closure success rates were 75.0% (*n* = 9) for the expert and 83.3% (*n* = 10) for the novice. No adverse events were reported.

**Conclusions:**

A small number of cases were required for both expert and novice endoscopists to reach competency in the novel through‐the‐scope suture device.

## INTRODUCTION

Endoscopic resection is a minimally invasive procedure for excising superficial gastrointestinal neoplastic lesions. The introduction of endoscopic submucosal dissection (ESD) has enabled the en bloc resection of tumors irrespective of their size and configuration, significantly reducing the risk of local recurrence.^1^ This technique has become a standard practice across Asian countries and is increasingly used in Western nations. The management of large post‐ESD mucosal defects remains challenging due to the associated risks of complications including delayed bleeding and perforations. Such complications can cause patient discomfort, extend hospital stays, and increase healthcare costs.^2^ This concern is exacerbated by the growing number of elderly patients and individuals on antithrombotic medications.^3^

Prophylactic endoscopic closures have been shown to reduce the incidence of delayed bleeding according to retrospective studies.^4^, ^5^ The X‐Tack suture system (Apollo Endosurgery) is a novel through‐the‐scope device that approximates the mucosal defect by inserting four helical tacks, connected by a polypropylene suture, into the surrounding tissue.^10^ Preclinical in vivo research and multicenter clinical studies have demonstrated its efficacy in endoscopic closure, achieving high success rates with reasonable procedure times across various target organs.^11^, ^12^, ^13^ However, the learning curve for both novice and experienced endoscopists using the X‐Tack system remains to be determined. We hypothesized that both expert and novice endoscopists can quickly become proficient in using the X‐Tack suture system to efficiently perform closures. The primary aim of our animal study is to determine the number of cases required for expert and novice endoscopists to achieve competency with this system.

## METHODS

### Participants

This study involved two endoscopists: a novice, with less than 400 experiences of upper gastrointestinal endoscopy and no experience in ESD, and an expert, with more than 500 experiences of ESD. Neither had previous experience with the X‐Tack suture system. Both endoscopists received training via instructional videos provided by Apollo Endosurgery 1 week prior to the experiment. The instructional videos detailed guidance on the operation of the device and technical advice for handling challenging situations such as device delivery issues and incorrect placement.

### Surgical preparation

In Japan, the X‐Tack suture system has not been approved yet. This study used an in vivo model to accurately simulate the dynamics of live tissues, such as tissue elasticity and blood circulation, which are critical for understanding the challenges of device manipulation in a real clinical setting. Four female pigs weighing between 41 and 46 kg (mean, 44 kg) underwent a 24‐h fasting period before the procedure. General anesthesia was administered through intramuscular injection of midazolam (0.2 mg/kg; Dormicum; Astellas Pharma Inc.) and medetomidine (40 µg/kg; Domitor; Nippon Zenyaku Kogyo Co., Ltd.), followed by intravenous propofol (2.0 mg/kg; Diprivan; AstraZeneca PLC). After tracheal intubation was performed, general anesthesia was maintained using isoflurane inhalation (1%–3%; Forane; Abbott Japan Co., Ltd.).

### Creation of mucosal defects

A therapeutic gastroscope (GIF‐Q260J; Olympus Medical Systems Co.) was inserted into the stomach, followed by the insertion of an overtube (Flexible Overtube; SB‐Kawasumi Laboratories, Inc.). The stomach was thoroughly lavaged with water. A mixture of hyaluronic acid solution (MucoUp; Boston Scientific) with 0.004% indigo carmine dye was injected to create a submucosal lift. A total of 24 mucosal defects, ranging from 2–4 cm in diameter, were generated with the EMR‐L technique with a ligation device (EBL device; SB‐Kawasumi Laboratories, Inc.) and snare (SD‐221Ls▪U‐25; Olympus Medical Systems Co.) in conjunction with a high‐frequency thermal coagulation device (VIO 3; Erbe Elektromedizin GmbH.). The size of each defect was confirmed with an endoscopic measuring device (Olympus Medical Systems Co.).

### X‐Tack suture system

**FIGURE 1:**
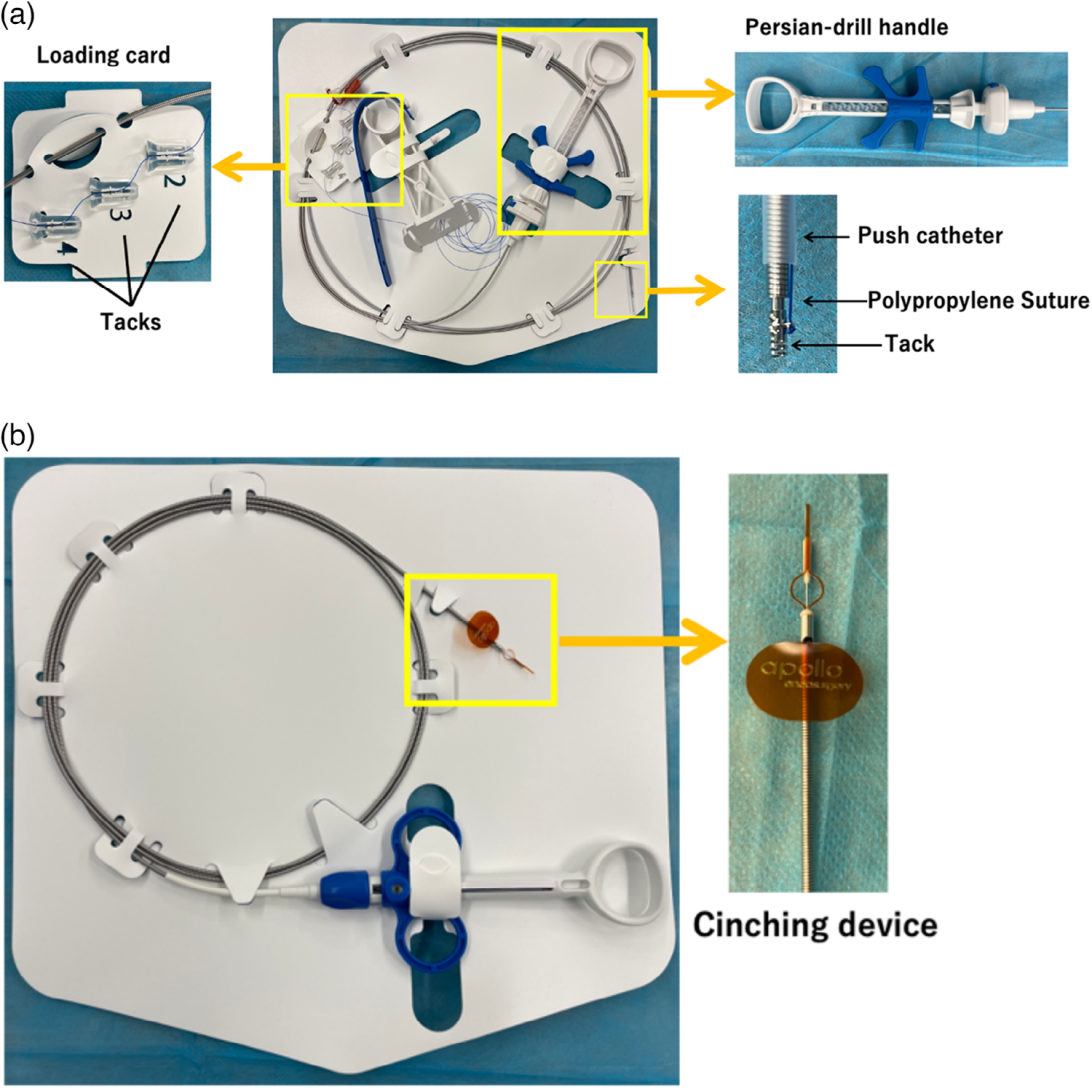
The X‐Tack device and suture cinch is used for the through‐the‐scope suture. The X‐Tack device comprises the push catheter with a tack connected to a polypropylene suture and a loading card with three additional tacks for reloading (a). A separate suture cinch is used to securely fix the suture (b).

The X‐Tack suture system consists of an outer catheter, four helical tacks, and a push catheter equipped with a Persian drill driver handle (Figure 1a). Each 5 mm helical tack is preloaded on a 3‐0 polypropylene suture threaded through an eyelet. First, the outer catheter is inserted into the accessory channel of the endoscope. The first tack is delivered into the gastrointestinal lumen using the push catheter, which anchors the tack into the tissue by rotating the inner catheter wire with the Persian drill handle to the appropriate depth. After deployment, the push catheter is withdrawn from the endoscope to be reloaded with the next tack from the loading card. The suture is progressively tightened with each tack placement. Once all four tacks are positioned around the mucosal defect, the push catheter is removed. A cinching catheter (Figure 1b) is then introduced to apply tension to the suture, approximating the edges of the defect together. The cinch is released, cutting and securing the suture in place.

### Endoscopic closure technique

**FIGURE 2:**
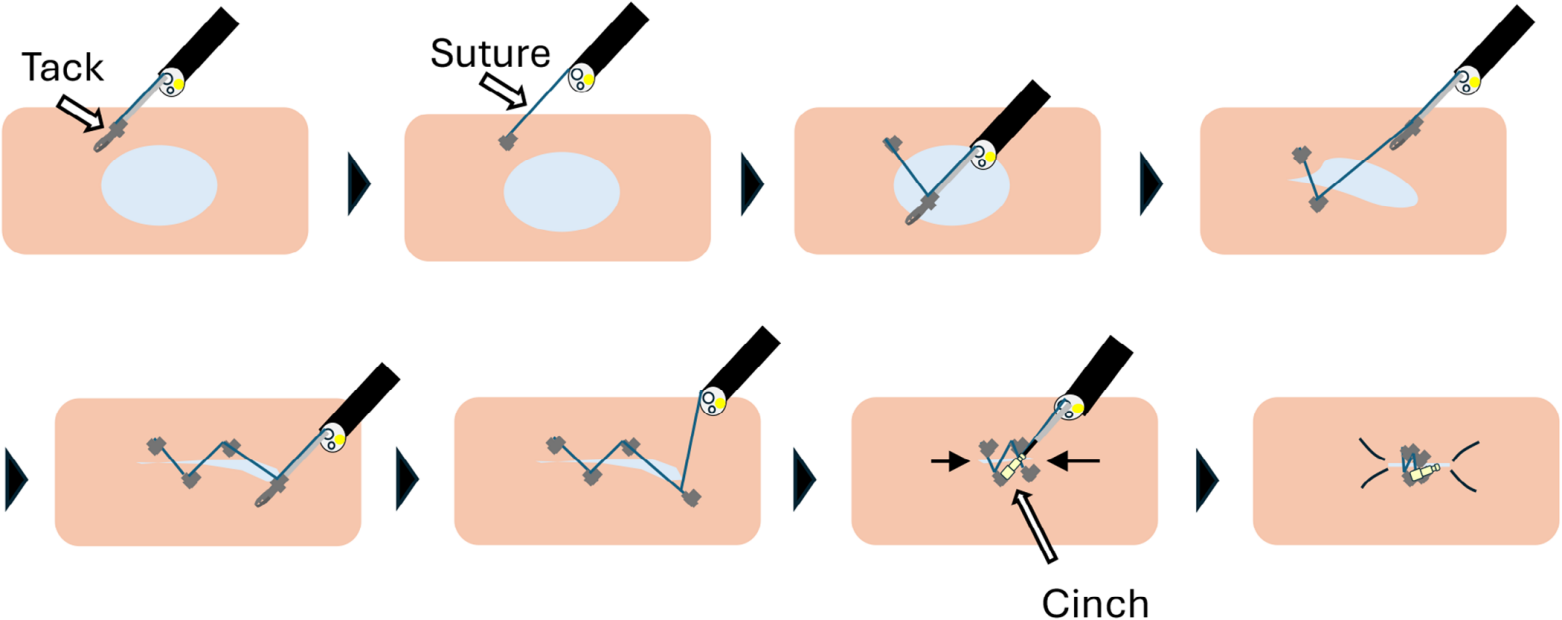
“Z‐shaped” closure with the X‐Tack system. The four tacks are consecutively embedded into the gastric wall, in a Z‐shaped manner. Finally, the cinch is applied to approximate and secure the four tacks.

Each endoscopist was randomly assigned 12 mucosal defects using a random number generator. They performed the endoscopic closure using the X‐Tack suture system following the standard protocol.^12^ For each closure, the four tacks were positioned in a Z‐shaped configuration around the defect and secured with a cinch (Figure 2). The procedural steps are demonstrated in Video S1. Each procedure was limited to the use of a single X‐Tack suture system, and any incompletely closed defects were not corrected with additional devices. After all procedures were finished, the pigs were immediately euthanized.

### Outcome measures

The primary endpoint was to determine the number of cases needed to reach competency, defined as completing the procedure in less than the literature‐reported average of 7.7 min.^11^ Incomplete closures were excluded from the analysis. Secondary endpoints included procedure times, complete closure rates, incidence of adverse events, and submucosal dead space appearance rates. Procedure time was measured from the insertion of the first tack to the placement of the cinch. Complete closure was defined as achieving over 90% closure of the mucosal defect, as verified by two certified endoscopy technicians reviewing the endoscopic images. Adverse events were defined as intraoperative complications such as bleeding or perforation that required additional intervention. The submucosal dead space appearance was assessed by endoscopic visualization after the procedure. Additional analyses included the comparison of procedure times between the endoscopist's first six and the latter six cases.

### Statistical analysis

In a preclinical porcine study, the use of the X‐Tack system for gastric mucosal defect closures reported an average procedure time of 7.7 min.^11^ In a multicenter cohort study, the procedure time of endoscopic closure for post‐colorectal ESD mucosal defect using the X‐Tack system resulted in an interquartile range of 6.3–17.3 min.^12^ Assuming an initial procedure time of 18 min and a learning rate of 10% per procedure, an endoscopist would need 8 cases to achieve the target time of 7.7 min. Considering the documented technical failure rate of 7.3%, we assigned 12 cases to each endoscopist to ensure sufficient opportunity to reach competency.

Descriptive data were presented as the median and interquartile range (IQR) or mean (±SD) for continuous variables and frequency counts (proportions) for categorical variables. Differences between groups were assessed using a *t*‐test or Mann‐Whitney U test for continuous variables and a chi‐squared test or Fisher's exact test for categorical variables. Statistical significance was set at *p* < 0.05. To determine the number of cases needed to reach competency, inverse curve fitting through nonlinear regression was conducted using the one‐phase decay equation model *Y* = (*Y*0 ‐ Plateau) × exp (‐K × *X*) + Plateau, with the number of cases as the independent variable (*X*), procedure time in min as the dependent variable (*Y*), and K represented as the rate constant. All statistical analyses and creation of graphical representations were performed using Prism version 9.5.1 (GraphPad Software).

## RESULTS

**FIGURE 3:**
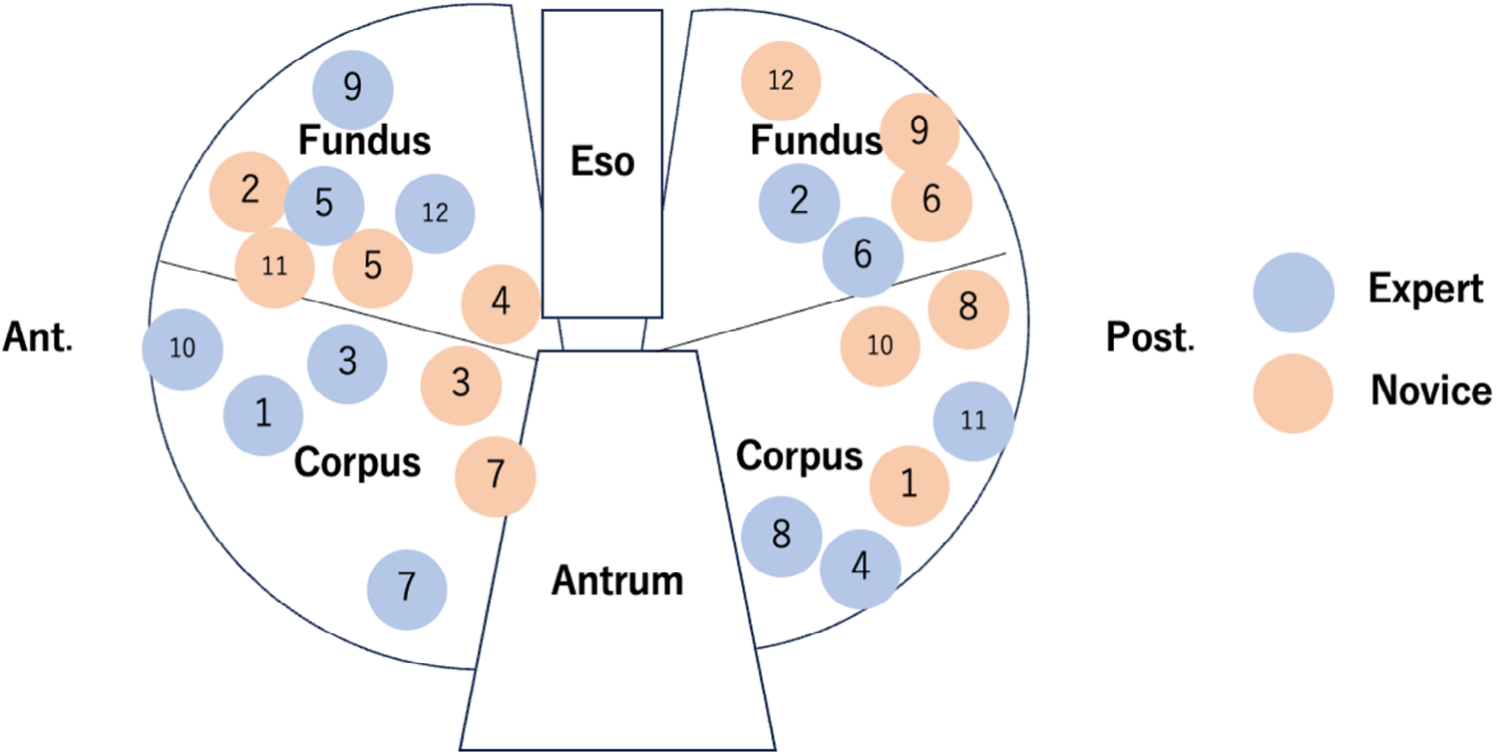
The distribution of mucosal defects in the porcine stomach. The blue circles represent the mucosal defects allocated to the expert, and the red circles represent the mucosal defects allocated to the novice. Eso: esophagus, Ant: anterior wall, Post: posterior wall

The distribution of mucosal defects allocated to each endoscopist is detailed as follows (Figure 3): the expert was allocated seven defects on the anterior and five on the posterior wall, whereas the novice was allocated six defects on both anterior and posterior walls. The mean diameter ± SD of the defects was 2.9 ± 0.2 cm (range 2.4–3.0 cm) for the expert, and 2.9 ± 0.2 cm (range 2.4–3.2 cm) for the novice, with no significant difference between the two (*p* = 0.857).

**FIGURE 4:**
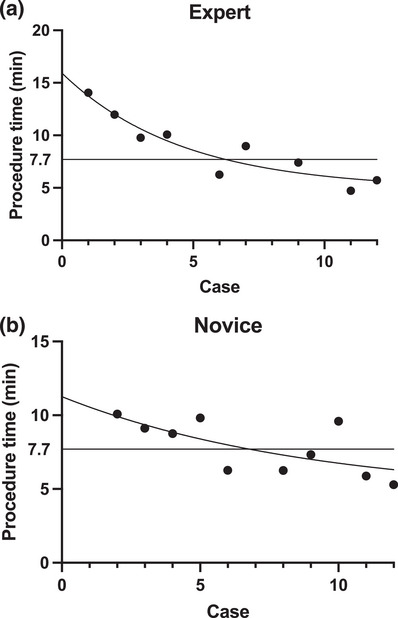
(a) Dot plot of procedure time for each case for the novice. The curve represents the inverse curve fitted in a one‐phase decay model. (b) Dot plot of procedure time for each case for the expert. The curve represents the inverse curve fitted in a one‐phase decay model.

Figure 4 illustrates the procedure time for each case, the fitted inverse curve, and the 7.7‐min benchmark. The expert endoscopist required six cases to reach proficiency, while the novice endoscopist required seven. The median closure time was 9.0 (IQR 6.0–11.0) min for the expert, and 8.0 (IQR 6.2–9.7) min for the novice, with no significant difference between them (*p* = 0.590).

**FIGURE 5:**
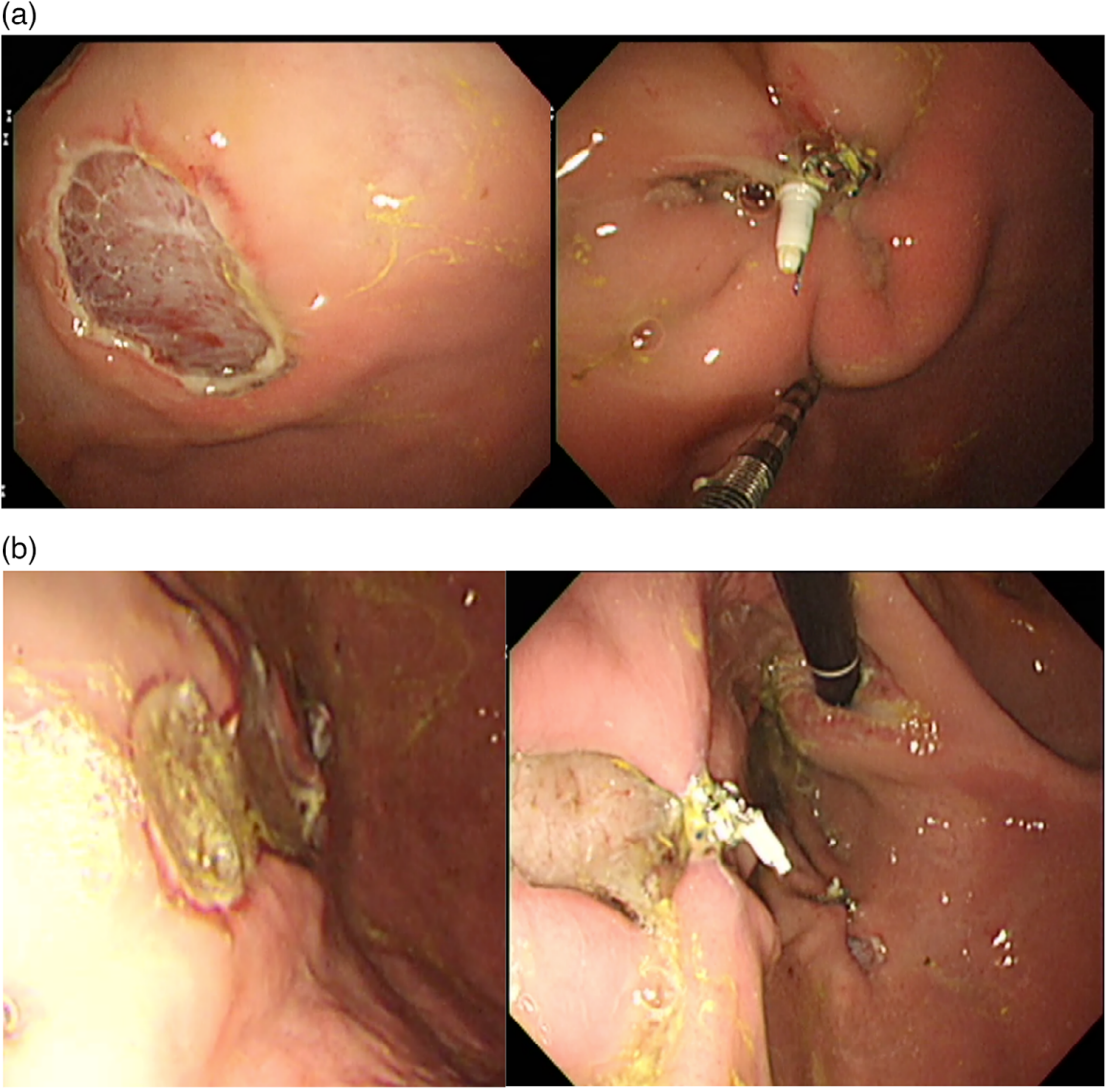
(a) Example of a successful endoscopic closure. The mucosal defect (left) is 30 mm in diameter. After endoscopic closure with X‐Tack, over 90% of the mucosal defect is closed (right). (b) Example of an insufficiently approximated mucosal defect. The mucosal defect (left) is 30 mm in diameter. After endoscopic closure with X‐Tack, less than 90% of the mucosal defect is closed with the formation of a submucosal tunnel (right).

Complete closure rates were 75.0% (*n* = 9) for the expert and 83.3% (*n* = 10) for the novice, with no statistically significant difference (*p* = 1.0). The expert encountered two cases of suture disconnection during cinching at the anterior and posterior walls of the greater curvature of the corpus. Additionally, there was one case where a suture formed an irreversible knot within the gastric cavity while closing a lesion on the anterior wall of the lesser curvature of the fundus. The novice experienced one incomplete closure due to a helical tack dislodgment within the gastric cavity while closing the lesion on the anterior wall at the lesser curvature of the corpus, and another case where more than 90% closure could not be achieved on the defect at the posterior wall of greater curvature of the corpus. Figure 5 provides an example of successful complete closure as well as a case where the mucosal defect was inadequately approximated. No adverse events occurred during the study. The appearance rate of submucosal dead space was 47.1% (*n* = 5) for the expert and 75.0% (*n* = 8) for the novice, with no statistically significant difference (*p* = 0.41). For the expert, the median closure times were 10.1 (IQR 8.0–13.0) min for the first six cases and 6.6 (IQR 5.0–8.6) min for the latter six cases (*p* = 0.064). For the novice, the median closure times were 9.1 (IQR 7.5–10.0) min in the first six cases and 6.3 (IQR 5.6–8.5) min in the latter six cases (*p* = 0.095; Table 1).

**TABLE 1:** Outcomes of the present study. Each endoscopist performed endoscopic closure with X‐Tack for 12 cases. SD: standard deviation, IQR: interquartile range.

	Expert	Novice	*p*‐value
Defect size; mean (SD); cm	2.9 ± 0.2	2.9 ± 0.2	0.866^*^
Cases needed to achieve competency; *n*	6	7	
Procedure time; median (IQR); min	8.2 (5.6–10.1)	8.0 (6.3–9.4)	0.86^**^
Complete closure success rate; % (*n*)	75.0 (9)	83.3 (10)	1.0^***^
Adverse event rate; %	0	0	1.00^***^
Submucosal dead space appearance rate; % (*n*)	41.7 (5)	75.0 (8)	0.41^***^

* *t*‐test.

** Mann‐Whitney *U* test.

*** Fisher's exact test.

## DISCUSSION

This animal research suggests that both novice and expert endoscopists can achieve proficiency with the X‐Tack suture system after only a small number of cases. The similar procedure times between the two operators demonstrate that the X‐Tack system is relatively easy to use for closing gastric mucosal defects and can be quickly mastered by operators with varying levels of experience.

The complete closure rates were lower than the previously reported X‐Tack studies.^11^, ^12^ This is likely due to endoscopists’ initial experience and the restriction of using only a single device for each closure, whereas previous reports allowed multiple X‐Tack devices or additional clips. In two cases, the suture was unintentionally severed due to excessive force during cinching, an issue previously noted in a multicenter study.^12^ Coordinating movements with the assistant to apply optimal suture tension might resolve this problem. Cases of tack dislodgement and knot formation resulted from improper tack reloading, requiring the procedure to be started over. Operators should take care to reload the tack correctly and prevent suture entanglement. In one case of incomplete closure, the initial two tacks were placed in the mid‐portion of the mucosal defect, which caused subsequent tacks to be disproportionately positioned on one side, leaving the opposite side open. In such a situation, an “X‐shaped” closure would have been more effective than a “Z‐shaped” closure.^10^ There was no specific tendency in the location of the mucosal defect that resulted in incomplete closure due to these mechanical issues. While our primary outcome focused on the number of cases needed to reach a specific procedure time, mechanical challenges continued to result in incomplete closures beyond this threshold, which is a limitation of our study.

Various methods to endoscopically close the mucosal defects have been investigated, which include TTS clips, over‐the‐scope systems, and suturing devices.^6^, ^7^, ^8^, ^9^ However, mucosal defect closure with these conventional methods is technically challenging and requires extensive procedure times. While TTS clip‐based closures are commonly used for small defect closures, advanced techniques such as the reopenable‐clip over‐the‐line method (ROLM) and the origami technique are necessary to address larger defects.^14^, ^15^, ^16^, ^17^, ^18^, ^19^ These strategies require multiple clips and expertise. Over‐the‐scope clips can only be used for defects of relatively small defects with a single chance for positioning.^20^ OverStitch (Apollo Endosurgery) system enables endoluminal full‐thickness suturing of large defects^8^; however, the bulky over‐the‐scope device faces limitations in accessing deep anatomical locations and operating within narrow working space. Endoscopic hand suturing involves complex techniques of precisely grasping and inserting the needle at the right angle.^9^, ^21^ For endoscopists in training, a device designed for quick and easy deployment is desirable for performing endoscopic closures. The X‐Tack system requires only basic endoscopic maneuvers, similar to those used in target biopsies. The four tacks can be placed at any desired location, offering the flexibility needed to manage moderately large mucosal defects. Furthermore, any misplacement of tacks can be corrected by simply reverse‐rotating the handle. The TTS delivery system enables access to lesions in various locations, including those requiring retroflex endoscopic positioning. As a result, the X‐Tack system provides a viable solution for endoscopists to address mucosal defects.

Recently, a reopenable, TTS single‐use clip with anchor prongs (MANTIS Clip; Boston Scientific) is now available. The MANTIS Clip grasps the edge of a mucosal defect by hooking the tissue with its prongs, drawing the tissue over to the opposing mucosal edge, and then reopening to approximate both sides before being deployed to secure the closure. In a case report, endoscopic closure of a 25 mm gastric defect using the MANTIS Clip demonstrated a closure time of approximately 7 min.^22^ Additionally, a case series on colorectal mucosal defects reported a median closure time of 7.9 min when using the MANTIS Clip to close post‐ESD defects of up to 61 mm in size.^23^ Meanwhile, the MANTIS Clip can be challenging to hold onto the thick mucosal edge during the reopening process, particularly in larger gastric defects, due to the increased tension of dragging the mucosa. In contrast, none of the tacks dislodged once embedded in the gastric wall with the X‐Tack system. Further investigations are warranted to clarify the differences in the learning curve, durability, and efficacy in closing mucosal defects across different sizes and various anatomical locations.

Our study did not evaluate the long‐term durability of the sutures. Submucosal dead spaces were observed in 13 out of 24 cases, although most of them were minor. There was no predilection for the location of the defect where the submucosal dead space formed. Previous research suggests that early dehiscence is associated with submucosal dead space, and placing sutures deeper within the submucosa and muscularis layers is essential for achieving durable closures.^24^ While we believe the X‐Tack system is suitable for initial approximation, additional sutures or clip‐based techniques may be required the minimize the submucosal space. Further investigations with a long‐term survival model are warranted to evaluate the durability of this device.

There are limitations to our study. The small sample size of endoscopists and the limited number of defects may limit the generalizability of the findings to the broader population. A larger sample size could yield different results. Additionally, the mucosal defects in our study ranged from 2.4 to 3.2 cm in diameter, so the applicability of our findings to defects larger than 3.2 cm is uncertain. Furthermore, the study was restricted to gastric mucosal defects, and the result may not be relevant for other anatomical locations, such as the duodenum or colorectum.

In conclusion, a relatively small number of cases were needed for both expert and novice endoscopists to become proficient in using the TTS suture device for endoscopic closure. This may be attributed to the operational simplicity of the X‐Tack, which minimizes the necessity for advanced technical skills in endoscopic maneuvers.

## CONFLICT OF INTEREST STATEMENT

Kazuki Sumiyama is a deputy editor‐in‐chief of DEN Open. The rest of the authors declare no conflict of interest.

## ETHICS STATEMENT

‐Approval of the research protocol by an Institutional Reviewer Board: N/A.

‐Informed Consent: N/A.

‐Registry and the Registration No. of the study: No.2019‐017.

‐Animal Studies: Ethical approval for the study protocol was obtained from the Institutional Animal Care and Use Committee of The Jikei University School of Medicine.

## Supporting information



Video S1 Endoscopic mucosal closure using the X‐Tack suture device.
