# Supplementary material for: The competency of the novel through‐the‐scope suture device for gastric mucosal defects: In vivo study in a porcine model (with video)
Source: DEN Open. 2024 Nov 12;5(1):e70037. doi: 10.1002/deo2.70037 (PMC11556257; doi:10.1002/deo2.70037)
Supplement: Supplementary file 1 — Video S1 Endoscopic mucosal closure using the X‐Tack suture device. [file DEO2-5-e70037-s001.docx]

Video link is available here:

[XTackProcedureVideo.mp4 - Google Drive](https://drive.google.com/file/d/1BBOWW6ib7hO2-b_EhpvwAKLzrAcV4DpU/view)
